# The Relationship Between Communicative Actions, Behavioral Intentions, and Corporate Reputation in the Framework of Situational Theory of Problem Solving in a Public Health Crisis

**DOI:** 10.3389/ijph.2023.1606301

**Published:** 2023-12-01

**Authors:** Eyyup Akbulut

**Affiliations:** Department of Public Relations and Publicity, Faculty of Communication, Atatürk University, Erzurum, Türkiye

**Keywords:** the situational theory of problem solving, communicative action, behavioral intention, corporate reputation, world health organization, infodemics, risk communication

## Abstract

**Objectives:** This study aims to determine the individuals’ communicative actions based on the basic assumptions of the situational theory of problem solving (STOPS) and the effect of these actions on people’s willingness to follow WHO’s instructions in the event of an epidemic. It also seeks to determine the impact of corporate reputation on people’s communicative actions and intention to follow instructions.

**Methods:** Data were collected digitally from 261 graduate students enrolled at a state university in the Eastern Anatolia Region of Turkey. A structural equation model (SEM) was employed for data analysis.

**Results:** Perceptual antecedents affected situational motivation, and situational motivation affected communicative actions. Communicative actions were a determining factor in individuals’ willingness to follow instructions. The perception of corporate reputation influenced both communicative actions and people’s willingness to follow instructions.

**Conclusion:** The study revealed that STOPS can provide an important theoretical framework for more effective risk communication practices in public health crises such as epidemics. It also displayed the relationship between the individuals’ communicative actions and their willingness to follow instructions and the determining effect of corporate reputation on both of these factors.

## Introduction

Pandemics remain a significant global concern with outbreaks occurring and spreading more quickly and easily in a tightly connected world. To prevent the spread of an outbreak, public health authorities should not only detect the extent of the outbreak and the risks it will pose but also take an increasingly active role in risk communication to ease heightened uncertainty and impede worldwide transmission. Therefore, risk communication is of vital importance in developing public health preparedness strategies [1].

There are many international studies on the management and prevention of health emergencies. Among these, the Sendai Framework (The Sendai Framework for Disaster Risk Reduction 2015–2030) attracts attention due to its holistic approach to disaster risk reduction. The Sendai Framework, supported by The United Nations Office for Disaster Risk Reduction (UNDRR) and announced in 2015, puts health risks and health resilience at the center of global disaster risk management [2]. The Sendai Framework categorizes epidemics and pandemics as a biological hazard [3] and highlights the critical role of risk information and communication in disaster risk management [4]. As a matter of fact, the COVID-19 pandemic, caused by the SAR-CoV-2 virus, hit all countries of the globe in 2019 and has once again shown how significant risk communication is in epidemic control. Many studies (e.g., [5–7]) report that risk communication has a positive effect on individuals’ display of protective and preventive behaviors.

To prevent excessive panic and anxiety and manage the pandemic scientifically and effectively [8], communication is one of the most crucial risk management tools during an epidemic [9]. Because when a public health crisis occurs, such as an epidemic, people find themselves in a state of uncertainty regarding the transmission and its potential health effects [10]. This uncertainty not only forces people to deal with a lot of questions but also fuels conspiracies and unfounded claims [11–14]. As stated in many studies (e.g., [15–19]), to avoid the uncertainty that might have such negative implications and to make the right decisions during an outbreak, individuals tend to seek information about the epidemic. Preventing epidemics or reducing the rate of transmission depends on individuals’ adoption of the behaviors necessary to protect themselves from the epidemic, their risk perceptions about the epidemic, their perception of trust in information sources, and their use of these sources. When individuals receive accurate and timely information about the epidemic, this directly affects their perceptions of risks and enables them to take the necessary measures to protect themselves [20]. In other words, during a public health crisis, individuals must be aware of the health hazards they face as well as the preventative steps they can take to protect their lives and health. People can make better decisions and take action to safeguard themselves, their families, and their communities from life-threatening health risks when timely, accurate information is made available to them in languages and media they can understand, trust, and use [21].

Effective public communication by health authorities is crucial in combating an epidemic since it encourages the public to respond appropriately to help contain the outbreak, limit exposure, and reduce morbidity and mortality [22]. For this reason, health authorities should carefully analyze the communicative behaviors of people during epidemic periods and the factors that affect these behaviors.

Although each state has its local health authorities in combating an epidemic, an effective global response has become increasingly important as epidemics quickly might turn into pandemics due to elevated levels of economic integration and trade, widespread travel, and rapid modes of transport [23]. For example, the COVID-19 disease was declared a pandemic by the World Health Organization (WHO) on 11 March 2020, just 3 months after WHO’s China Country Office released a statement regarding “viral pneumonia” cases in Wuhan, China, on 31 December 2019. WHO plays a leading role in coordinating responses to epidemic-prone diseases such as yellow fever, cholera, Ebola virus disease, Zika virus, influenza, and coronavirus, and supporting national, regional, and international efforts to prevent and mitigate epidemics. In addition to an online learning platform (OpenWHO), WHO has numerous emergency and risk communication guidelines, reports, and training modules featuring expert opinions and experience gained from previous outbreaks (e.g., [21, 24–26]). However, the availability of a large number of documents or applications provided by WHO does not guarantee that people will use them as a source of information and follow its instructions. Therefore, this study seeks to answer to what extent people will be willing to obtain and transmit the information provided by WHO and follow WHO’s instructions in a public health crisis. Thus, the study tests a theoretical model to reveal what kind of communicative actions individuals might take in a public health crisis such as an epidemic within the framework of STOPS and investigates the effect of these actions on the behavior of following WHO instructions. The model also examines how public perception of WHO’s corporate reputation influences people’s communicative actions and intention to follow WHO directions.

### The Situational Theory of Problem Solving

The situational theory of problem solving (STOPS) is an extended and generalized version of the situational theory of publics [27]. STOPS, proposed by Kim and Grunig [27], is a communication theory that explains why and how individuals communicate in a problematic situation [28]. STOPS assumes that “the more one commits to problem resolution, the more one becomes acquisitive of information pertaining to the problem, selective in dealing with information, and transmissive in giving it to others.” [27].

STOPS has three independent variables, perceptual, cognitive, and motivational antecedents, and a dependent variable called communicative actions. Problem recognition, the first of the three perceptual antecedents, refers to people’s perception that something needs to be done about a problem and the extent to which they stop and think about what to do, while the second perceptual antecedent, constraint recognition, refers to people’s perceptions that there are barriers that limit their ability to do something about the problem. The final perceptual antecedent, involvement recognition, describes how people perceive the connection between the problematic situation and themselves [29]. Another antecedent (independent variable) within STOPS is situational motivation. Situational motivation refers to the situation-specific cognitive and epistemic readiness for one’s problem-solving efforts, in the sense of reducing the inconsistency between the expected situation and the experienced situation [27].

According to STOPS, when individuals perceive a certain situation/issue as a problem, associate the situation with themselves, and perceive that there are few or no constraints limiting them in solving the problem, they will be more motivated to engage in active communicative actions to obtain a solution.

Considering the previous studies on the effect of these three independent perceptual variables on situational motivation, this study proposes the following hypotheses:

H1 Problem recognition of individuals positively affects their situational motivation.

H2 Involvement recognition of individuals positively affects their situational motivation.

H3 Constraint recognition of individuals negatively affects their situational motivation.

Communicative actions, included as an independent variable in STOPS, can be separated into three groups: information acquisition, information selection, and information transmission. Each of these three communicative actions is then divided into two based on activity and passivity, thus defining a total of six communicative actions. Information acquisition, the first of these communicative actions, includes two behaviors: information seeking (active) and information attending (passive). While information attending means reaching and processing any information concerning the problem in an unplanned manner, information seeking involves an individual scanning the environment to gather more detailed knowledge about the situation [27]. The second of the communicative actions, information selection, is divided into two groups: information forefending (active) and information permitting (passive). In information forefending, individuals fend off certain information in advance by judging its value and relevance for a given problem-solving task. On the other hand, in information permitting, individuals accept any information related to a problem-solving task [27]. Information transmission, the final of the communicative actions, includes two behaviors: information forwarding (active) and information sharing (passive). Information forwarding is the act of someone voluntarily sharing information with others about a problem, even if that information does not provide a solution or is not requested by others [30]. In information sharing, individuals share information only when they are solicited [27].

According to STOPS, both situational motivation and referent criterion have a decisive effect on an individual’s communicative actions in a problematic situation. The referent criterion, which is acknowledged as a cognitive antecedent, is defined as “any knowledge or subjective judgmental system that influences the way in which one approaches problem-solving” [27], and it is associated with the circumstance in which an individual recalls their experiences in the context of problems that are similar to the current one while formulating a strategy to obtain a solution.

To determine the relationship between situational motivation, referent criterion, and communicative actions, this study posits the following hypotheses:

H4 Situational motivations of individuals positively affect their communicative actions.

H5 The referent criterion positively affects the communicative actions of individuals.

### Behavioral Intention

Intentions include motivational factors that affect behavior and are indicators of how much effort people are willing to put in to perform the behavior [31]. The literature typically discusses behavioral intention in the context of planned behavior theory. Planned behavior theory claims that the stronger the intention to engage in a behavior, the more likely should be its performance [31]. In epidemic control, it is crucial that individuals follow the instructions from recognized institutions and organizations. People must, however, intend to act in this way for it to happen. The role of communication in forming behavioral intention in the context of STOPS has been the subject of numerous studies. For instance, Yan et al. [32] found that information forwarding, one of the main communicative actions in STOPS, had a greater impact on behavioral intention than information seeking.

On the other hand, Pressgrove, Barra, and Janoske [33] demonstrated that information permitting, sharing, and attending, which are accepted as passive communicative actions in STOPS, are more effective than active communicative actions in terms of intention to donate, take time to volunteer, and participate in policy advocacy. Yoo, Kim, and Lee [34] revealed that information acquisition mediated both the relationship between intention and the selection and transmission of information as well as the impact of perceived risk on intention. In their study of organic food consumers in Australia, Sultan et al. [35] showed that perceived communication, satisfaction, and trust improve the intention- behaviour gap and perceived behavioural control-behaviour gap in the framework of planned behavior theory. Xu, Li, and Shan [36] reported that information selection and information acquisition may affect people’s intention to get the HPV vaccine, while information transmission does not have such an effect. And Chon and Park [37] demonstrated that information transmission and acquisition affect people’s intentions to follow the CDC’s guidelines in a public health emergency.

Based on these literature findings, this study proposes the following hypothesis:

H6 Communicative actions of individuals positively affect their willingness to follow WHO’s instructions.

### Corporate Reputation

Corporate reputation is the overall assessment of stakeholders about an organization based on their direct experience with the organization over time, any other communication and symbolism that provides information about the organization’s actions, and comparisons of the organization with the actions of its leading competitors [38].

Kim [39], in his study examining the perceptions and communicative actions of publics in crisis communication, found that there is a positive relationship between communicative actions (information attending, information forwarding, and information seeking), reputation, and behavioral intention.

In their public segmentation study through an analysis of the communicative actions of individuals in a crisis, Kim, Miller, and Chon [40] reported that aware and active publics are more likely to have negative behavioral intentions toward the organization.

The following hypotheses were developed in light of these studies.

H7 Individuals’ perceptions of WHO’s reputation positively affect their communicative actions.

H8 Individuals’ perceptions of WHO’s reputation positively affect their behavioral intentions.

**FIGURE 1:**
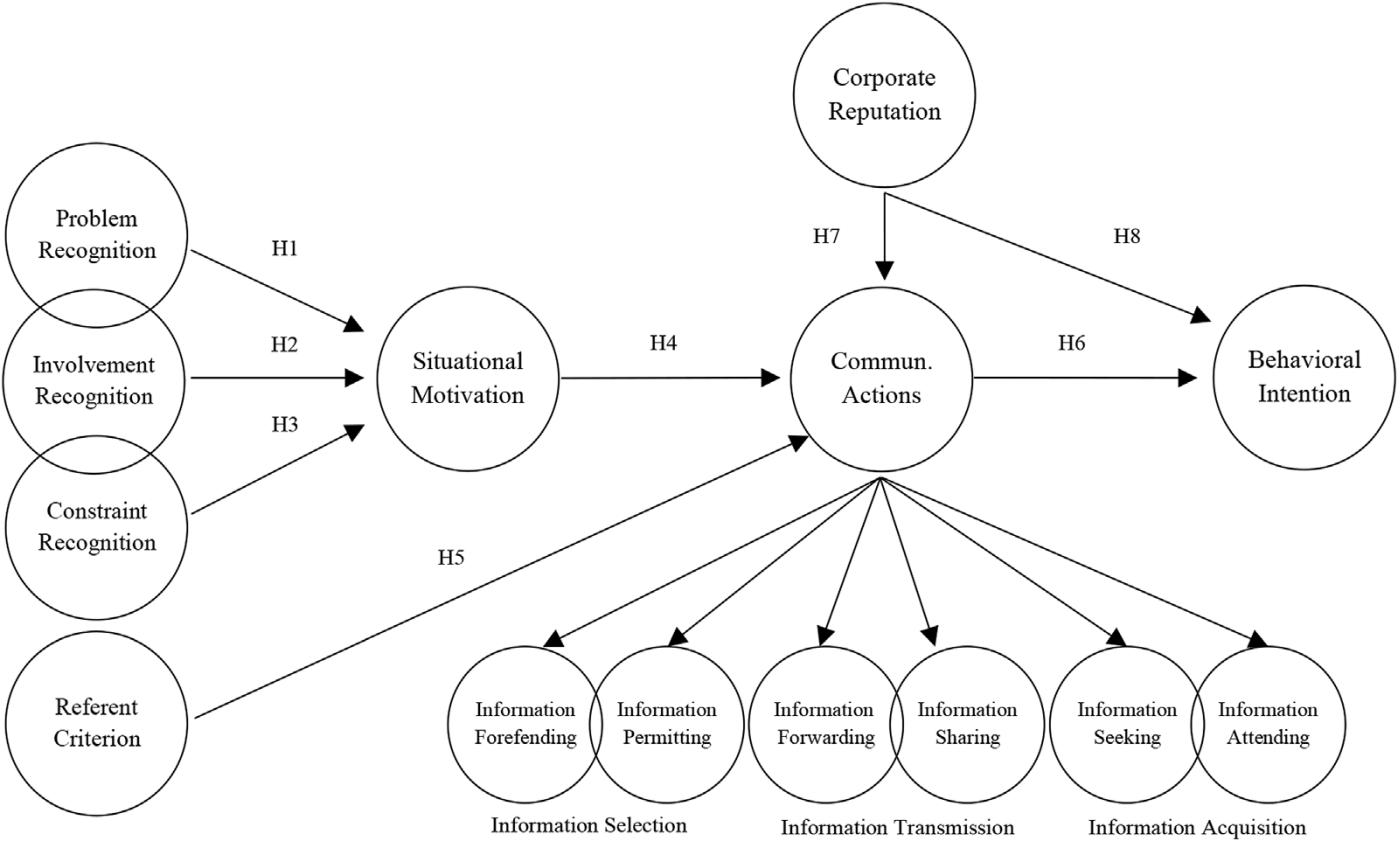
Proposed research model and hypotheses (Erzurum, Türkiye. 2022).

Figure 1 presents the model proposed in this study.

## Methods

### Research Design and Sampling

The data were collected between November and December 2022 using an online survey technique. First, a questionnaire form was created on Google Forms, and then the link to the questionnaire was sent to the participants via e-mail and WhatsApp. The questionnaire was examined by three field experts to ensure that the language use and expressions in the scales were appropriate.

The questionnaire consisted of five sections in total. In the first section, there were three questions about the demographic characteristics of the participants. The second section included fifteen items about situational variables. The third section featured eighteen items related to information behaviors. The fourth section was about behavioral intention and presented three items. The last section included six items related to corporate reputation.

The research population consisted of graduate students from a state university located in the Eastern Anatolia Region of Turkey. The data were collected from a total of 294 participants who have the power to represent this population by using the convenience sampling method, which is one of the non-probability sampling methods. However, 33 questionnaire forms were excluded due to non-compliance with the analysis suitability criteria; therefore, the analysis was carried out on 261 questionnaire forms. After reading and signing a consent form outlining the study’s objectives, participants completed the online questionnaire.

### Measures

In this study, the main variables of STOPS were measured through a total of 33 items, three for each variable, reflecting similar studies in the literature [27, 37, 41, 42]. A six-item scale, established by Sarstedt and Schloderer [43] to measure the reputation of non-profit organizations, was used to ascertain the participants’ perceptions of the corporate reputation of WHO. Chon and Park’s [37] 3-item scale was employed to determine participants’ behavioral intentions to follow WHO’s instructions. A 5-point Likert-type scale ranging from 1 (I strongly disagree) to 5 (I totally agree) was used for all items.

### Analysis

The proposed model was tested using structural equation modeling (SEM) with the AMOS v20 program. Before testing the model, a series of confirmatory factor analyses were performed to check the low or cross-loaded items and the validity of the measures used in the study. The analysis results displayed that the measurement scales generally provided a satisfactory internal consistency as indicated by Cronbach’s α scores (see Supplementary File S1). To establish the factor structure of communicative actions, a second-order confirmatory factor analysis was also carried out.

The measurement model was determined compatible with the model fit indices in the acceptable range [44–47]: X^2^(738) = 1,389.98, *p* < .001; X^2^/df = 1.883; CFI = .933; GFI = .799; IFI = .934; TLI = .922; RMSEA = .058; SRMR = .052.

## Results

The demographic characteristics of participants are shown in Table 1.

**TABLE 1:** Participants characteristics (Erzurum, Türkiye. 2022).

Variable	Category	n	%
Gender	Male	129	49.4
	Female	132	50.6
Age (years)	21–30	170	65.1
	31–40	65	24.9
	≥41	26	10.0
Educational level	Master’s student	209	80.1
	Doctoral student	52	19.9

The participants were 50.6% (*n* = 132) female and 49.4% (*n* = 129) male. While 65.1% (*n* = 170) of the participants were aged between 21–30 years, 24.9% (*n* = 65) were aged between 31–40 years, and 10.0% (*n* = 26) were aged 41 years or older. Finally, 80.1% (*n* = 209) of the participants were educated at the master’s level and 19.9% (*n* = 52) at the doctoral level.

After determining that the fit indices of the measurement model were within the acceptable range, the structural model was created and tested. As a result of the path analysis, the regression weights of the variables were examined and as a result of the analysis, it was seen that the path between the referent criterion and communicative actions was not statistically significant (*β* = .08, *p* > .05) and therefore H5 was rejected. Based on the findings of a similar study in the literature [37] and considering the controversial position of the referent criterion within STOPS [27], it was removed from the model and the structural model was tested again.

**FIGURE 2:**
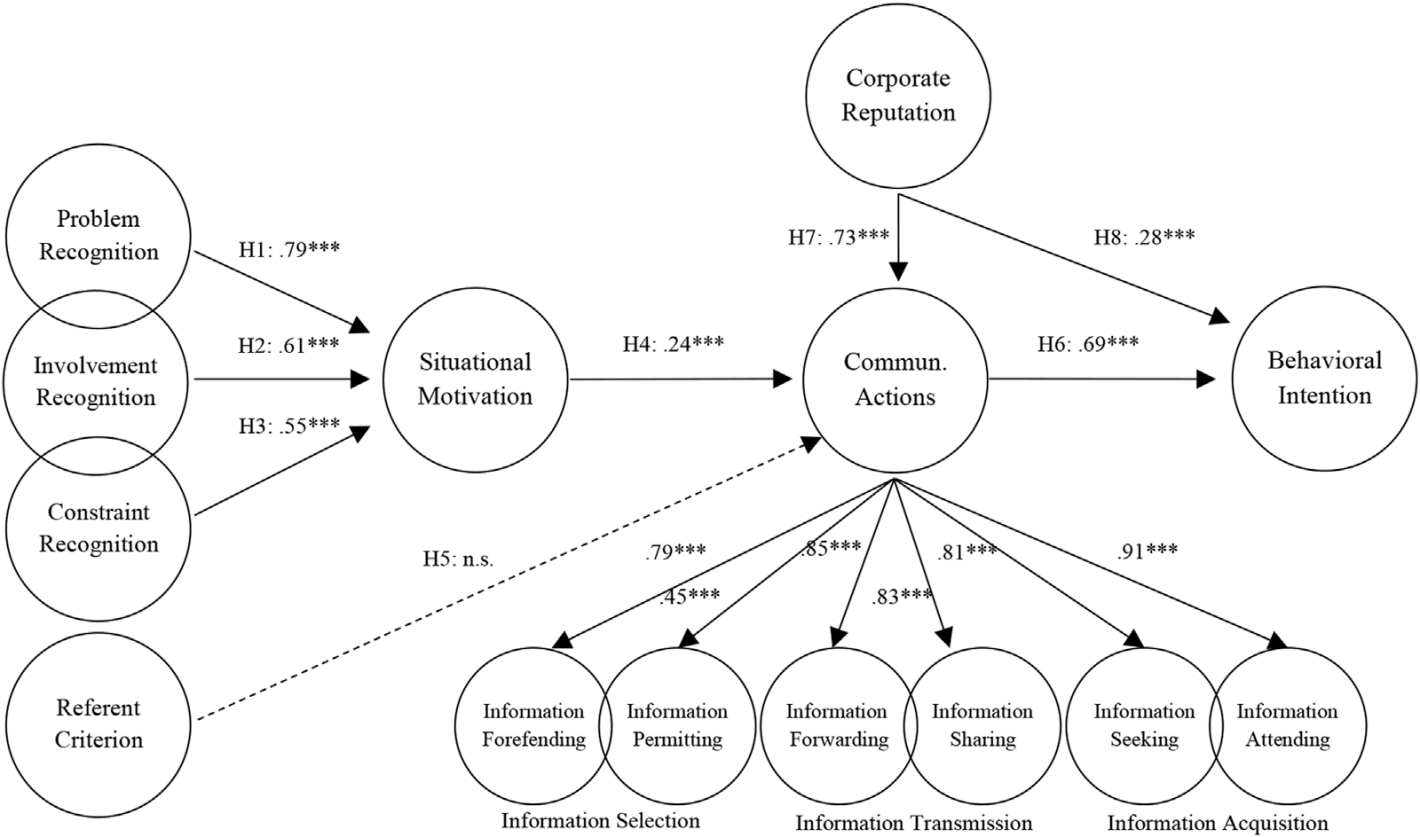
The results of testing the structural model. Standardized solutions for significant paths in structural equation modeling analysis are reported (***p < 0.001) (Erzurum, Türkiye. 2022).

Data model fit criteria were used to evaluate the proposed model. The results indicate that the fit indices of the model were in an acceptable range [44–47]: (X^2^(309) = 607.731, *p* < .001; X^2^/df = 1.967; CFI = .952; GFI = .859; IFI = .952; TLI = .945; RMSEA = .061; SRMR = .054). The findings related to the model are summarized in Figure 2.

The first five hypotheses of this study aim to test the basic assumptions of STOPS, which is used to predict when and what kind of communicative actions individuals will exhibit in the event of a public health crisis. Among these hypotheses, H1, H2, and H3 are related to three perceptual antecedents that affect individuals’ situational motivations for a particular problem. The results confirm that H1, expressed as “Problem recognition of individuals positively affects their situational motivation,” was accepted (*β* = .79, *p* < .001). H2, which predicts that individuals’ involvement recognition positively affects their situational motivation, was also accepted (*β* = .61, *p* < .001). This study used reversed items to evaluate H3, which is expressed as “Constraint recognition of individuals negatively affects their situational motivation.” Therefore, the path between the variables should be positive for the H3 hypothesis to be accepted. According to the results, H3 was accepted (*β* = .55, *p* < .001). H4 predicted that the situational motivations of individuals about the problem would have a positive effect on their communicative actions. According to the model’s results, H4 was accepted (*β* = .24, *p* < .001). H6, expressed as “Communicative actions of individuals positively affect their willingness to follow WHO’s instructions,” was accepted (*β* = .69, *p* < .001). Finally, H7 and H8 proposed that corporate reputation will have a positive effect on individuals’ communicative actions and behavioral intentions, respectively. The findings indicate that both H7 (*β* = .73, *p* < .001) and H8 (*β* = .28, *p* < .001) were accepted.

## Discussion

The results confirm the basic assumptions of STOPS, except for the referent criterion, and are consistent with similar studies in the literature (e.g., [27, 37, 41, 42, 48]). In a public health crisis, individuals’ situational perceptions affect their situational motivations, and their situational motivations have a decisive effect on their communicative actions. In other words, the findings demonstrate that when individuals recognize the problem, believe that there are no obstacles to taking action on the problem, and think the problem is relevant to themselves, they will be more motivated to engage in communicative behaviors such as acquiring, selecting, and transmitting WHO information. In addition, individuals are found to prefer more active communicative actions (information forefending and information forwarding) in both information selection and information transmission behaviors. In this process, individuals first select information that can contribute to the solution of the problem (information forefending). They then voluntarily transmit this information to others, even if they are not asked to do so (information forwarding). Thus, they contribute to faster and more effective dissemination of reliable information. Therefore, as stated by Kim and Hong [42], public health authorities, especially WHO, should increase individuals’ problem recognition by strongly emphasizing that the current situation can pose a serious threat to their health through traditional and new media in public health crises. In addition, public health authorities should try to positively change the perception of constraints and obstacles in solving the problem by clearly explaining how individuals can contribute to the solution and state that the problem can seriously affect individuals and their relatives. Because sharing scientific information with the public instantly and rapidly is an effective way to reduce panic that may occur [49].

One of the important results of the study was that the referent criterion, which is the cognitive variable of STOPS, did not have a significant effect on communicative actions. In a similar previous study [37], this result is justified by the fact that participants did not perceive a link between themselves and a hypothetical epidemic. This explanation can be valid for this study, as well. This study attempted to determine the communicative actions of individuals in the event of an unreal, hypothetical epidemic, and participants could not be expected to have sufficient knowledge regarding a specific and unrealistic outbreak. In fact, the place of the referent criterion in STOPS is often controversial. Although the referent criterion was included in the early versions of the situational theory of public, which is the previous version of STOPS, it was removed from the model in later studies on the grounds that it could not adequately explain communicative actions [27]. Nevertheless, it should not be overlooked that in the event of a real and specific public health crisis, the referent criterion may have a decisive influence on the communicative actions of individuals. A study conducted by Kim and Hong [42] on COVID-19, which is a real and specific pandemic, concluded that individuals who have sufficient knowledge about the COVID-19 pandemic engage in various communicative actions. For this reason, health authorities should also produce documents and content that will enable individuals to obtain sufficient information about epidemics in risk communication studies.

Similar to previous studies (e.g., [32, 33, 36, 37]), this study also revealed that individuals’ communicative actions have a decisive influence on behavioral intention (the intention to follow WHO’s instructions). Therefore, health authorities should actively and sufficiently inform individuals, especially on the most critical elements of the epidemic, release resources containing up-to-date information, and create easily accessible communication channels for individuals who seek to obtain more detailed information. Individuals with reliable and sufficient information will thus be more motivated to follow the instructions of the health authorities and will share the information they have obtained with others.

Another question examined within the study’s purview is whether corporate reputation influences both communicative actions and behavioral intention. The effect of corporate reputation on communicative actions and behavioral intentions has not received much attention in the literature. The findings from this study demonstrated that individuals’ opinions about the WHO’s corporate reputation have a positive effect on their communicative actions in case of a public health emergency. In other words, individuals are more likely to actively acquire, select, and transmit information offered by WHO when they have a positive perception of the reputation of WHO.

This study also concludes that favorable perceptions of WHO’s reputation have a positive effect on individuals’ behavioral intentions (to follow WHO’s instructions). The willingness of individuals to engage with health authorities during epidemic periods and the acceptance of the recommendations of these authorities are strongly influenced by the individuals’ perceptions of honesty, trustworthiness, and competence [50]. Public trust in health authorities and in the information disseminated by these authorities is a critical factor in people’s adherence to preventive measures during epidemics [51–53]. Trust plays a vital role in dealing with the infodemic. Because trusted sources are more likely to influence people. But there is no trust without trustworthiness. Therefore, health authorities should strive to gain the trust of individuals by acting honestly and transparently and providing a continuous flow of information from the beginning of the outbreak [54]. In a public health crisis, individuals’ perceptions of the trustworthiness of health authorities in their ability to address the risk or crisis are based on the preexisting reputation of these authorities, the quality of their past and current relationships with the individuals, and the reliability of the information they provide [50]. For this reason, health authorities, especially WHO, should focus more on relationship management to build long-term and strong relationships with their stakeholders, on reputation management to foster a positive reputation, and on more effective risk communication planning, even when there is no immediate public health crisis. Relationship and reputation management and risk communication are organizational functions that have a critical role in building corporate trust. These three functions and corporate trustworthiness have a vital impact on the effectiveness of the leadership required for the prevention and control of epidemics [50].

### Limitations and Future Research

This study which demonstrates that STOPS offers a useful framework for effective risk communication in a public health crisis and provides a theoretical basis for more comprehensive and detailed researches has some important limitations. First, it employs a cross-sectional design. Future studies with a longitudinal design may provide more information in evaluating the results of this study. Second, only graduate students were included in the sample. In addition, the sample was selected using the non-probability sampling method. Therefore, the generalizability of the findings is limited. In future studies, a probability sampling method may be chosen, and it may be preferable to use a larger sample representative of different social groups. Third, the study was based on a possible epidemic scenario, and a specific epidemic was not selected. Therefore, individuals’ responses may differ in the event of an actual or specific epidemic, as indicated in the context of the referent criterion. Fourth, only a small number of variables are included in the model. Future studies might employ more variables that may affect individuals’ communicative actions and behavioral intentions, such as source or message reliability. Finally, it is possible to investigate whether behavioral intention affects both active and passive communicative actions.

### Conclusion

In public health crises such as epidemics, it is crucial for individuals to display appropriate communicative actions and adhere to the instructions of health authorities to help prevent or control the outbreak. In this context, as a result of the study, it was revealed that STOPS, which provides a comprehensive explanation of when and how individuals communicate, and whose basic assumptions are confirmed, except for the referent criterion, offers a useful framework for effective risk communication. The study also contributed to the literature on STOPS and risk communication by incorporating corporate reputation and behavioral intention variables into the model. The finding that corporate reputation has positive effects on individuals’ communicative actions and their willingness to follow instructions has indicated that health authorities, particularly the WHO, should invest more in corporate reputation management. The study’s findings are expected to provide academics and public health experts with valuable insights into communicative actions of individuals in the event of a public health crisis.
