# Supplementary material for: The Relationship Between Communicative Actions, Behavioral Intentions, and Corporate Reputation in the Framework of Situational Theory of Problem Solving in a Public Health Crisis
Source: Int J Public Health. 2023 Dec 1;68:1606301. doi: 10.3389/ijph.2023.1606301 (PMC10721679; doi:10.3389/ijph.2023.1606301)
Supplement: Supplementary file 1 [file Table1.docx]

Supplementary Material

**Supplementary File 1.** Factor loadings, means, standard deviations and Cronbach's α values (Erzurum, Türkiye. 2022)

| Variable | Measurement items | Item loading | Cronbach’s α | Mean (SD) |
| --- | --- | --- | --- | --- |
| Problem Recognition |  |  | .73 | 3.80 (.99) |
|  | PR1 | .74 |  |  |
|  | PR2 | .82 |  |  |
|  | PR3 | .90 |  |  |
| Involvement Recognition |  |  | .94 | 2.10 (1.05) |
|  | IR1 | .90 |  |  |
|  | IR2 | .92 |  |  |
|  | IR3 | .93 |  |  |
| Constraint Recognition |  |  | .83 | 3.48 (.90) |
|  | CR1 | .84 |  |  |
|  | CR2 | .77 |  |  |
|  | CR3 | .77 |  |  |
| Referent Criterion |  |  | .85 | 3.32 (.77) |
|  | RC1 | .81 |  |  |
|  | RC2 | .89 |  |  |
|  | RC3 | .75 |  |  |
| Situational Motivation |  |  | .88 | 3.30 (.84) |
|  | SM1 | .86 |  |  |
|  | SM2 | .83 |  |  |
|  | SM3 | .82 |  |  |
| Information Forefending (.76)^1^ |  |  | .86 | 3.48 (.99) |
|  | IFF 1 | .76 |  |  |
|  | IFF2 | .89 |  |  |
|  | IFF3 | .81 |  |  |
| Information Permitting (.68) |  |  | .81 | 3.77 (.75) |
|  | IP1 | .85 |  |  |
|  | IP2 | .72 |  |  |
|  | IP3 | .71 |  |  |
| Information Forwarding (.86) |  |  | .83 | 3.44 (1.00) |
|  | IFO1 | .64 |  |  |
|  | IFO2 | .89 |  |  |
|  | IFO3 | .88 |  |  |
| Information Sharing (.86) |  |  | .85 | 3.49 (1.02) |
|  | ISH1 | .91 |  |  |
|  | ISH2 | .74 |  |  |
|  | ISH3 | .69 |  |  |
| Information Seeking (.83) |  |  | .95 | 3.11 (1.24) |
|  | ISE1 | .95 |  |  |
|  | ISE2 | .96 |  |  |
|  | ISE3 | .89 |  |  |
| Information Attending (.90) |  |  | .90 | 3.60 (1.07) |
|  | IA1 | .90 |  |  |
|  | IA2 | .87 |  |  |
|  | IA3 | .86 |  |  |
| Behavioral Intention |  |  | .92 | 3.59 (1.09) |
|  | BI1 | .86 |  |  |
|  | BI2 | .91 |  |  |
|  | BI3 | .89 |  |  |
| Corporate Reputation |  |  | .93 | 3.38 (1.00) |
|  | CRP1 | .65 |  |  |
|  | CRP2 | .83 |  |  |
|  | CRP3 | .85 |  |  |
|  | CRP1 | .86 |  |  |
|  | CRP2 | .87 |  |  |
|  | CRP3 | .89 |  |  |
| ^1^Scores in parenthesis after information forefending, information permitting, information forwarding, information sharing, information seeking, and information attending represent first-order factor loadings. PR= Problem Recognition; IR= Involvement Recognition; CR= Constraint Recognition; RC= Referent Criterion; SM= Situational Motivation; IFF= Information Forefending; IP= Information Permitting; IFO= Information Forwarding; ISH= Information Sharing; ISE= Information Seeking; IA= Information Attending; BI= Behavioral Intention; CRP= Corporate Reputation. | | | | |
